# Analyzing the perception of happiness among Korean medical students using a concept mapping methodology: a cross-sectional study

**DOI:** 10.3389/fpubh.2025.1476022

**Published:** 2025-03-19

**Authors:** Jaemu Lee, Kyung Hye Park, Sangmi Teresa Lee

**Affiliations:** ^1^Department of Computer Education, Busan National University of Education, Busan, Republic of Korea; ^2^Department of Medical Education, Yonsei University Wonju College of Medicine, Wonju, Republic of Korea; ^3^Department of Emergency Medicine, Yonsei University Wonju Severance Christian Hospital, Wonju, Republic of Korea

**Keywords:** happiness, quality of life, multidimensional scaling analysis, concept mapping, medical students

## Abstract

**Introduction:**

Happiness differs according to population groups and cultures. For medical students, more studies have focused on negative emotions than on happiness. This study explored the overall perceptions and standards of medical students to analyze the concept of happiness from various perspectives in the Korean context.

**Methods:**

A concept mapping analysis comprising five stages was conducted with medical students at Yonsei University’s Wonju College of Medicine in South Korea. Focus questions were generated in Phase 1, and 23 students participated in individual brainstorming in Phase 2. Fifty statements were confirmed in Phase 3. Sixteen students assigned an importance score to each statement and participated in the individual sorting of statements and naming of categories in Phase 4. Finally, the concept maps were interpreted using multidimensional scaling and hierarchical cluster analysis.

**Results:**

The medical students’ perception of happiness was divided into two dimensions, “Study–Life” on the X-axis and “Self–Relationship” on the Y-axis, and was expressed in three categories and five sub-categories. The subcategories of “Self-management” and “Quality of life” were grouped under “Personal development,” “Social support” was named as a single category, and “Guaranteed future” and “Academic achievement” were grouped as “Professional fulfillment.” The most important sub-category for medical students was “Social support.” Among the statements generated in these categories, the most important was “When I have a healthy body and stamina,” which belonged to “Quality of life.”

**Discussion:**

This study showed that to enhance the happiness of medical students, a system that supports their social relationships, careers, learning, and individual efforts is required. The results of this study can provide information for the development of student support programs that allow medical educators and institutions to promote medical students’ happiness.

## 1 Introduction

Happiness is difficult to define in a single word, and its definition may change depending on the context. It is sometimes used interchangeably with quality of life, subjective well-being, and life satisfaction and is highly correlated with these concepts. In previous studies, happiness has been defined as an individual’s overall assessment of their quality of life according to their own standards (1, 2), which differ by individual. The perception of happiness varies across different cultural contexts and academic disciplines among university students (3). For example, American and Korean college students perceived their happiness differently (4, 5), while medical students demonstrated lower psychological well-being scores compared with non-medical students (6).

Quantitative studies on negative emotions such as stress, burnout and depression are sufficiently reported in previous studies (7–9). For instance, in a systematic review, Rotenstein et al. reported that the prevalence of depressive symptoms among medical students was 27.2% (10). However, studies on the overall perception of happiness targeting medical students are lacking, leaving happiness a positive and relatively underexplored domain.

Tools such as the Oxford Happiness Questionnaire (OHQ) have been widely used to measure happiness; however, this is a one-dimensional scale consisting of items on positive and negative emotions, life satisfaction, and happiness characteristics (1, 11). Therefore, since respondents only answer the items given in the survey, other concepts of happiness remain difficult for researchers to identify.

The happiness of medical students is likely to involve unique contextual factors. In fact, the rigorous academic workload, evaluation systems, and identity formation process as future professionals may be critical factors defining their happiness. However, more research needs to address the overall perception and concept of happiness among medical students. China, Korea, and Japan are representative of nations with a confusion culture (12). While studies exist on mental health and positive psychology among Chinese medical students (13, 14), there is limited research on positive psychological aspects such as happiness among medical students in Japan and Korea. As Korean researchers, we recognized the necessity of studying happiness among Korean medical students. Although we found recent research, most was restricted to analyzing only the correlation between the educational environment in medical schools and happiness, their study shows limitations in defining and exploring happiness as a multidimensional concept (15).

By employing concept mapping analysis to identify and derive the concepts and criteria of happiness that medical students perceive, this study aims to overcome the limitations of previous quantitative survey-based research. The conceptual mapping analysis is a desirable research method for exploring specific phenomena based on the subjective experiences of participants in fields that lack a pre-established theoretical system (16, 17). We believe that it is possible to analyze medical students’ perception of happiness by examining the concept of happiness. Therefore, the purpose of this study was to identify medical students’ concepts and standards of happiness using concept-mapping analysis. These results can provide an opportunity for medical educators and institutions to better understand the happiness of medical students and implement appropriate student support programs.

This study’s specific research questions are follows. First, what is happiness as perceived by medical students? Second, what do medical students think is the relative importance of happiness?

## 2 Materials and methods

### 2.1 Study design

This single-center, cross-sectional study explored the concept of happiness among medical students. Concept mapping analysis is a structured conceptualization process that explores and describes the hidden side of specific phenomena. It allows researchers to objectively understand the perception or concept of a phenomenon in a specific group by visually representing it (18).

### 2.2 Ethical considerations

This study was approved by the Institutional Review Board of the Yonsei University Wonju Christian Severance Hospital (CR322034). Participation was voluntary, and all of the participants provided informed written consent.

### 2.3 Participants

The participants of this study were medical students at Yonsei University’s Wonju College of Medicine. As for inclusion criteria, we selected medical students who had been in medical college for at least one semester, who understood the purpose and methods of the study, and who voluntarily agreed to participate in the study. Responses that could not be included in the research data analysis because they did not agree to participate in the study or were insincere were excluded. Because it is possible for the participants who generate the ideas and those who sort and rate them to be different when using this methodology (19).

Participants were recruited separately for these two phases. In a concept mapping study, research participants should be opportunistically sampled in a potential conceptual universe, considering heterogeneity.

### 2.4 Sample size

Although there is no limit to the number of research participants in a concept mapping study, more than ten participants are required to fully generate ideas (18). In previous studies using concept mapping, the number of participants has varied from 20 to 629 in the brainstorming stage, depending on the research theme (20). However, as medical students voluntarily participated in the brainstorming stage in this study, we determined that response collection would be complete when the saturation of the description of the concept of happiness was confirmed. In the next step (sorting and rating), approximately 15 participants are recommended (21). In a review of concept mapping studies, the average number of participants in this stage was approximately 14 (20). Therefore, we planned to recruit 16–17 participants in this study, considering that 10% of the participants may drop out.

### 2.5 Data collection

The concept mapping research procedure proposed by Kane and Trochim was used in this study (18). Participants were categorized by sex and grade.

First, in the preparation step, the following focus questions were created to collect students’ thoughts on the concepts of happiness. Two authors (JL and KHP) made the questions to stimulate the general happiness concepts of the students.

- Who is a happy medical student?
- What do you need for a happy medical school life?
- Describe a specific experience in which you felt happy after becoming a medical student.

Second, ideas were generated. Medical students were asked to freely express their thoughts on the focus questions using a questionnaire hosted on SurveyMonkey. The survey link was listed on a recruitment notice that was posted on a bulletin board in the medical college building, which students could access if they wished to participate. Responses to the survey were collected between June and September 2022. Sixty statements were generated after qualitative analysis using the participants’ ideas (JL and KHP).

Third, statements were structured. All of the researchers evaluated the statements, classified them into temporary categories. Each statement should contain one idea that does not overlap with another. All participants were able to accurately understand the statements’ contents. All statements were expressed in short phrases, such as “When I do…” or “When I am…” Finally, 50 statements were confirmed.

Fourth, in the representation step, the medical students who rated the statements and created a similarity matrix were recruited via another notice in the medical college building. They visited one of the researchers’ offices where they received an orientation on how to rate statements and create similarity matrix tables. The rating and sorting of statements took place in October and November 2022. The participants grouped similar statements into the same group and named them accordingly. The importance of each statement was indicated on a 5-point scale (1 = relatively unimportant, 5 = extremely important).

### 2.6 Instruments

To guide the generation of ideas during the preparation step, three open-ended focus questions were developed. As a next step, 50 concise statements were finalized and structured as short phrases for subsequent rating and grouping. Participants rated the importance of these statements on a 5-point Likert scale and grouped similar statements into categories, creating a similarity matrix essential for the concept mapping process. During the representation step, this task was facilitated through manual sorting and rating forms.

### 2.7 Data analysis

As continued representation step, the researcher (JL) created a group similarity matrix table by combining the similarity matrix tables evaluated by the medical students. A multidimensional scaling analysis was performed on the group similarity matrix. In the multidimensional scaling analysis, the number of dimensions was determined by considering three criteria: agreement, interpretability, and efficiency. Agreement represents the difference between the similar data evaluated by each interview participant and the finally derived spatial drawing, the degree of which is expressed as a stress value (18). Interpretability identifies whether the expressed dimensions are interpretable based on the attributes and contents of the statements (18). Efficiency determines the number of simple and of few dimensions as possible in consideration of agreement and interpretability (18). Kane and Trochim recommended that the concept map be expressed in two dimensions for efficient interpretation (18); therefore, this study also set it to two dimensions considering these results comprehensively. A distribution map based on the two-dimensional Euclidean distance model was derived. In concept mapping, statements located close to each other in two dimensions are recognized as having similar meanings, whereas statements far from each other are recognized as having different meanings. Hierarchical cluster analysis was conducted using the two-dimensional coordinate values obtained from the multidimensional scaling analysis. For cluster analysis, the Ward method was employed; as it makes distance-based data meaningful, hierarchical cluster analysis for conceptual mapping analysis is known to be particularly suitable (18). The number of clusters was then determined using a dendrogram, and concept mapping was completed by connecting the points corresponding to the statements within the clusters marked to the points from the multidimensional scaling analysis with lines.

Fifth, the concept mapping results were interpreted. As a result of the multidimensional scaling analysis, cluster names and dimensions were determined based on the relative positions of the statements displayed on the X- and Y-axes and the contents of the statements connected to the categories. Cluster names were determined based on the dendrogram results. The average importance and stress of the statements were calculated for each category. Finally, in the utilization phase, the overall results of the study were expressed as a concept map. Based on the results of the concept mapping, groups were identified and their names determined.

To analyze the qualitative content of the medical students’ ideas about happiness and create the statements, Microsoft Excel ver. 2018 (Microsoft Corp., Redmond, WA, United States) was used. IBM SPSS ver. 24.0 (IBM Corp., Armonk, NY, United States) was used for the statistical analyses.

## 3 Results

### 3.1 Study participants

A total of 23 medical students responded to the focus questions on the concept of happiness. Sixteen medical students participated in statement rating and constructing the similarity matrix table (Table 1).

**Table 1:** Study participants.

Demographics		Generation of ideas	Sorting and rating
Sex	Male: Female	10:13	10:6
Grade	Premedical 1st year	8	6
	Premedical 2nd year	2	3
	Medical 1st year	1	2
	Medical 2nd year	3	3
	Medical 3rd year	4	1
	Medical 4th year	5	1
Total		23	16

### 3.2 Concept mapping analysis for the perception of happiness

The 2D stress value was 0.279, which was within the average range (0.205–0.365) for multidimensional scaling analysis of concept mapping (22). The stress and squared correlation (RSQ) showed a relatively high explanatory power of 0.71.

**Figure 1:**
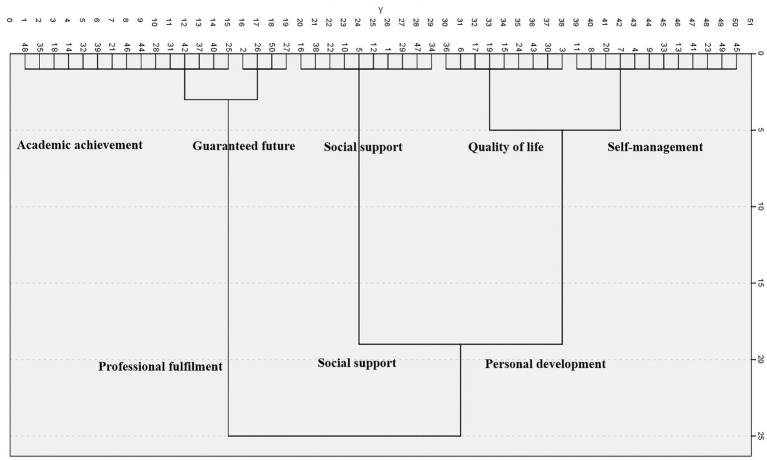
Dendrogram based on cluster analysis (Ward).

The number of clusters was 2–5 based on the hierarchical cluster analysis dendrogram of the individual statements located at the coordinates derived through the multidimensional scaling analysis. As a result of the multidimensional scaling analysis, the statements were analyzed and classified into three categories and five subcategories, as reflected in the dendrogram (Figure 1).

**Figure 2:**
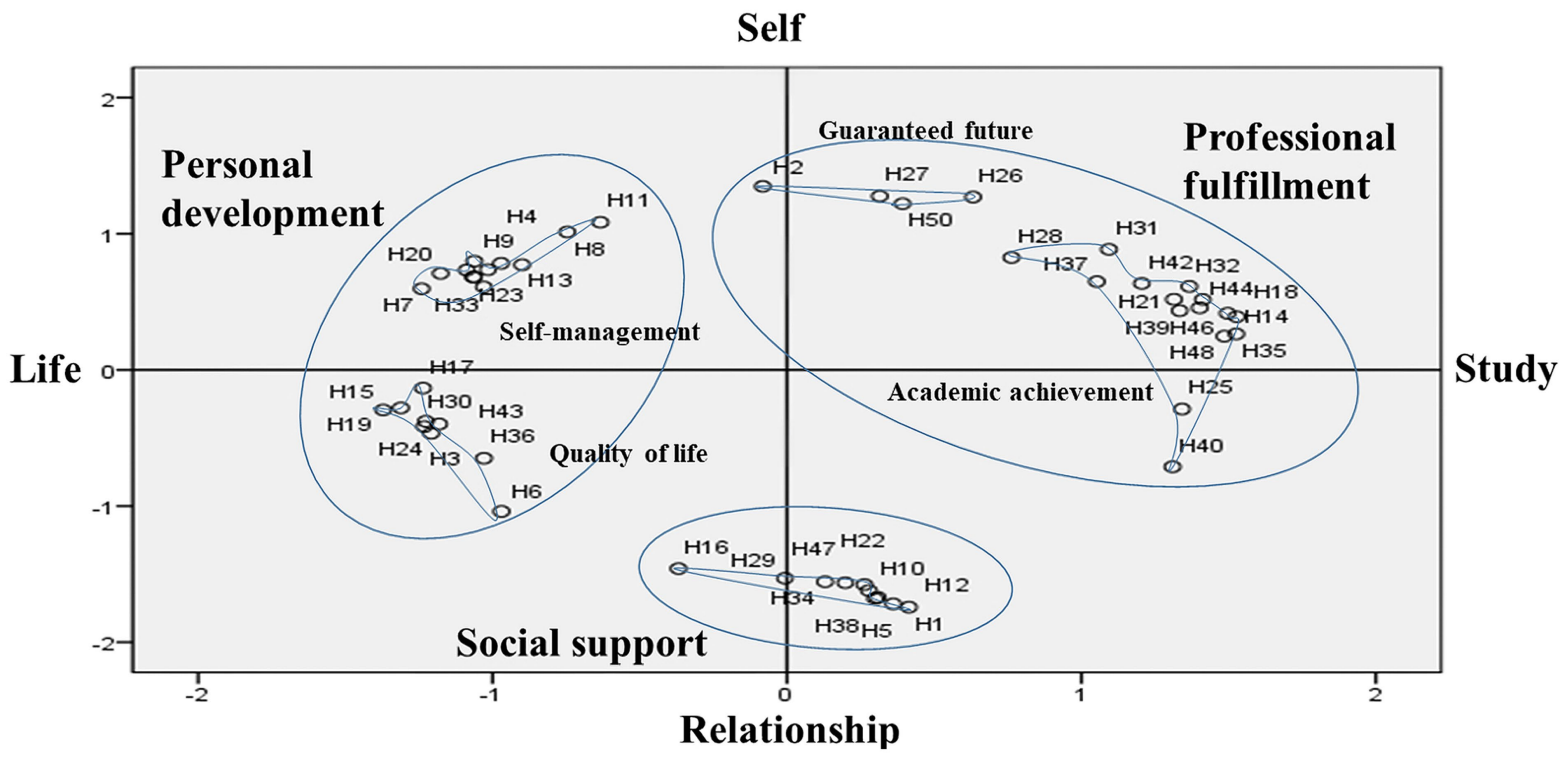
Concept map of when medical students feel happy.

The clusters derived from the hierarchical cluster analysis were determined by considering the relative positions of the statements displayed on the coordinates and the content of the statement bound to the category. The X-axis was named “Study–Life,” and the Y-axis was named “Self–Relationship.” The X-axis was divided into happiness related to academic achievement and personal life, and the Y-axis was divided into happiness with oneself and one’s relationships with others (Figure 2, Table 2).

**Table 2:** Statements located at the extreme end of the X-Y axis.

X-axis: study–life	
(+) Study	(−) Life
14. When I enjoy studying medicine (1.5274) 35. When I have safely passed difficult subjects without being flunked (1.5259) 18. When I feel that I am studying what I want to do (1.4966) 48. When I get a good grade compared to my work (1.4854) 44. When I feel proud and rewarded for my studies (1.4120) 46. When I feel that studying medicine is right for me (1.4017)	15. When I exercise (−1.3718) 19. When I have a healthy body and stamina (−1.3116) 7. When I have my own way to overcome stress (−1.2408) 17. When I maintain a good work–life balance (−1.2362) 3. When I am free to enjoy my hobbies (−1.2317) 30. When I get enough sleep every day (−1.2266)

Y-axis: self–relationship	
(+) Self	(−) Relationship
2. When I work hard and do not obsess over the results (1.3487) 27. When I have clearly defined my life goals (1.2764) 26. When I have a desired direction of life after graduation, including a career as a doctor (1.2701) 50. When I do not feel stressed about finding a job because a job (as a doctor) is guaranteed (1.2208) 11. When I am in control of my own life (1.0838)	1. When I have good friendship (−1.7428) 12. When I have friends I can share my feelings with (−1.7189) 10. When I get support from my family (−1.6792) 5. When I have friends to overcome studying difficulties with (−1.6706) 38. When I adapt to school in the process of meeting and getting to know new people (−1.6203)

Based on the results of the Euclidean distance model and the dendrogram of the cluster analysis, the categories were named “Personal development,” “Professional fulfillment,” and “Social support.” “Personal development” was classified into two sub-categories, “Self-management” and “Quality of life.” “Social support” was its own category. “Professional fulfillment” was classified into two sub-categories, “Guaranteed future” and “Academic achievement” (Table 3). “Self-management” included 12 statements about one’s attitude, emotions, life control, and self-awareness, while “Quality of life” included nine statements about one’s health, sleep, economic power, rest, exercise, and work-life balance. “Social support” included 10 statements about friends, quality of people around oneself, family, and social activities. “Guaranteed future” included four statements about one’s career as a doctor and their future goals, and “Academic achievement” included 15 statements about grades, studies, and examinations.

**Table 3:** Clusters and statements about “when medical students feel happy.”

Category	Subcategory (importance score)	Statement	Importance score
Personal development	Self-management (3.83 ± 0.35)	8. When I do my best in what I want to do	4.31 ± 0.79
		41. When I feel I am taking good care of myself	4.31 ± 0.70
		7. When I have my own way to overcome stress	4.25 ± 0.77
		45. When I feel good about myself and believe in myself	4.13 ± 0.96
		4. When I strive for self-realization even when I am busy	3.94 ± 1.12
		11. When I am in control of my own life	3.94 ± 1.12
		49. When I have an optimistic and positive personality	3.63 ± 1.45
		33. When I know what I like and what I do not like	3.56 ± 1.26
		20. When I am in control of my mood	3.56 ± 1.09
		23. When I have a non-comparison attitude	3.50 ± 1.21
		9. When I do not regret the past	3.44 ± 1.46
		13. When I know how to use my time effectively	3.44 ± 1.41
	Quality of life (3.82 ± 0.48)	19. When I have a healthy body and stamina	4.56 ± 0.73
		3. When I am free to enjoy my hobbies	4.31 ± 0.95
		30. When I get enough sleep every day	4.13 ± 1.26
		6. When I have more than enough money to live on	3.94 ± 1.29
		43. When I get enough rest on the weekend	3.94 ± 1.12
		15. When I exercise	3.50 ± 1.26
		17. When I maintain a good work–life balance	3.44 ± 1.26
		36. When I feel comfortable in my living space and study space	3.30 ± 1.26
		24. When I go on an overseas trip and experience a difference from my daily life	3.19 ± 1.38
	Social support (3.84 ± 0.49)	12. When I have friends I can share my feelings with	4.38 ± 0.89
		34. When I am evaluated as a trustworthy person for one reason or another by my peers	4.31 ± 0.79
		1. When I have a good friendship	4.31 ± 0.60
		5. When I have friends to overcome studying difficulties with	4.25 ± 0.86
		29. When I am in a happy relationship with a girlfriend/boyfriend	4.00 ± 1.10
		10. When I get support from my family	3.69 ± 1.08
		16. When I enjoy doing what I like while doing club activities	3.62 ± 1.02
		22. When I share my thought with like-minded people	3.44 ± 1.26
		38. When I adapt to school in the process of meeting and getting to know new people	3.44 ± 1.03
		47. When I participated in extracurricular activities (clubs, student council, etc.) at school	2.94 ± 1.00
Professional fulfilment	Guaranteed future (3.42 ± 0.57)	27. When I have clearly defined my life goals	4.00 ± 0.82
		26. When I have a desired direction in life after graduation, including a career as a doctor	3.69 ± 1.08
		2. When I work hard and do not obsess over the results	3.31 ± 1.35
		50. When I do not feel stressed about finding a job because a job (as a doctor) is guaranteed	2.69 ± 1.35
	Academic achievement (3.30 ± 0.43)	39. When I feel a sense of accomplishment with good exam results	3.94 ± 1.00
		44. When I feel proud and rewarded for my studies	3.89 ± 0.89
		18. When I feel that I am studying what I want to do	3.81 ± 1.33
		37. When I feel that medical knowledge is mine	3.69 ± 1.08
		42. When I can continue learning with interest in my current study	3.56 ± 0.81
		25. When I finish the examination	3.25 ± 1.44
		28. When I think that I can use what I have studied to serve many people	3.31 ± 1.25
		21. When I have an appropriate desire for grades	3.31 ± 1.01
		31. When I have decided on the field I want to major in as a resident	3.31 ± 1.01
		46. When I feel that studying medicine is right for me	3.13 ± 1.36
		14. When I enjoy studying medicine	3.13 ± 1.15
		32. When I have a clinical clerkship in the department I am interested in	3.00 ± 0.97
		35. When I have safely passed the difficult subjects without being flunked	2.94 ± 1.06
		48. When I get good grades compared to my work	2.60 ± 1.40
		40. When I discuss academic content with colleagues	2.56 ± 1.21

Importance scores are presented as mean ± standard deviation.

### 3.3 Importance of clusters and statements

Regarding the average importance score for each sub-category, “Social support” (3.84 ± 0.49) was rated as the most important, followed by “Self-management” (3.83 ± 0.35), “Quality of life” (3.82 ± 0.48), “Guaranteed future” (3.42 ± 0.57), and “Academic achievement” (3.30 ± 0.43). “When I have a healthy body and stamina” was considered the most important statement regarding happiness (4.56 ± 0.73), while “When I discuss academic content with colleagues” was rated as the least important (2.56 ± 1.21) (Table 3).

## 4 Discussion

In this study, concept mapping analysis was used to examine medical students’ perception of happiness. The analysis was structured using multidimensional scaling and hierarchical cluster analyses. As a result, the 50 statements related to students’ perception of happiness were divided into two axes (Study–Life and Self–Relationship) three categories (Personal development, Social support, and Professional fulfillment), and five sub-categories (Self-management and Quality of life; Social support; and Guaranteed future and Academic achievement).

The first cluster, “Self-management,” included medical students’ positive attitudes toward life, self-compassion, emotional control, mindfulness, and control over life, which was similar to previous studies showing that mindfulness training reduced medical students’ anxiety, stress, and depression and improved their coping styles, empathy, and happiness (23, 24). Self-compassion has also been associated with authentic and durable happiness among Chinese college students (25). Stress and burnout are common among medical students due to excessive learning and frequent exams. Similar to Zhang et al.’s study, where positive psychology education enhanced the psychological well-being of Chinese medical students (14), medical schools should provide courses or programs that can improve students’ emotional control, encourage mindfulness, and enhance their feelings of happiness.

Medical students said that they felt happy with their “Quality of life,” which includes rest, hobbies, sleep, exercise, health, and economic power. Among the 50 statements, “When I have a healthy body and stamina,” which had the highest importance score, belonged to this cluster. The importance students place on their physical health in this study can be found in previous research as well. Existing studies found similar factors influencing medical students’ happiness, including well-being, quality of life, and health (1, 7, 8, 11). In addition, good physical, mental, and social health, strong social ties, and sufficient sleep were also associated with life satisfaction among medical students (26). Several studies conducted among medical students in Cyprus, India, and China have demonstrated a positive correlation between high levels of physical activity and happiness (27–29). This suggests that medical schools should make medical students aware of the importance of physical activity for their happiness, and support the creation of a physical environment and time to promote physical activity.

“Social support” includes friends, parents, social activities, and self-evaluations. Social support had the highest importance score of the five clusters, and the inclusion of family members seemed to reflect the cultural characteristics of South Korea. Psychological, health, life satisfaction, and positive emotional scores were correlated with happiness and quality of life of New Zealand college students, but there was little correlation between social and environmental factors, and happiness and quality of life (1). However, in this study, social aspects were important. In an exploratory factor analysis of survey responses on happiness among American college students, emotional, psychological, and social factors were classified, but Korean college students had other factors such as family relations, financial means, religion, appearance, health, and leisure (4). Koreans value social belonging, and Korean adults considered belonging to be more important for happiness than social recognition (30). In a study examining Hofstede’s cultural dimensions across medical students and trainees from 16 countries, most Asian countries showed lower individualism scores compared with English-speaking countries, South American countries, and Israel. Korean medical students and trainees, like the general Korean adult population, demonstrated low individualism scores and emphasized collectivism, suggesting they place high value on group belonging (31). Studies conducted on Korean nursing students also highlighted the importance of social relationships, demonstrating that activating group dynamics enhanced subjective happiness, leading to improved self-efficacy and resilience (32).

Another interpretation is that medical students value relationships in closed social environments because they spend most of their time in medical schools or hospitals. In an Iranian study, medical students with secure attachment styles scored higher on the Oxford happiness inventory (33). In a Korean study using the Dundee Ready Educational Environment Measure (DREEM), students’ social self-perception (SSSP) was the only predictor of subjective happiness both before and after the COVID-19 pandemic (34). Medical school life is tough, but programs, counseling, and mentoring can help medical students maintain positive relationships and feel a sense of belonging. Medical students should attempt to build positive relationships with their families and friends to increase their happiness.

“Guaranteed future” refers to one’s stable career as a doctor and their life goals after graduation. A regression analysis showed that Iranian medical students’ level of happiness is related to their attitudes toward their studies, especially their field of study, and future careers (35). Similar to our study, postgraduate and master’s degree students were happy when their career optimism was higher (36). Students in health-related disciplines in Vietnam tend to report higher levels of subjective well-being, which may be attributed to their guaranteed employment prospects after graduation (37). Therefore, support is needed so that medical students can develop a positive attitude toward their studies, careers, and general future life direction.

“Academic achievement” involved grades and examinations. This cluster had the largest number of statements, indicating that students felt happiness related to academic achievement in various ways. However, this cluster also had the lowest importance score and it contained the statement “When I discuss academic content with colleagues,” which was also rated the least important. Academic motivation was significantly associated with subjective well-being among Vietnamese university students, regardless of their major (37). Although academic motivation is associated with happiness, in our study as in others, medical students’ grades did not seem to have a significant effect on their happiness. Yoo et al. reported that grade point average had no effect on Korean medical students’ subjective happiness (15). Medical students with low academic performance have higher happiness scores than those with high academic performance (38). This suggests that students’ grades have a low correlation with happiness and that whether they pass the course in general is more important.

The results of our study are consistent with those of a recent study that found that medical students were happier during the clinical practice period than during the pre-medical period (15). In addition, the medical students in this study felt happy about studying medicine and proud that their intended profession helped people. Enhancing medical students’ pride and reminding them of the importance of the medical study process without over-emphasizing the importance of their grades can also improve their happiness. Although high or low grades do not have a significant impact on happiness, they cannot be ignored. It is also worth considering the introduction of an assessment system that prioritizes course completion on a pass/non-pass basis rather than an assessment system that assigns grades. For example, Korean medical students with criterion-referenced assessments in the medical curriculum had lower scores for stress, burnout, and depression and higher scores for quality of life than those with norm-referenced assessments (39).

### 4.1 Limitations and strengths of the study

This study has a few limitations. As it was conducted at a single medical college in Korea, generalization of the results may be difficult. Small sample size and relatively subjective interpretation, due to research design, could also cause difficult generalization. Future research should generalize the results with the increased sample size, expansion to a multi-center study, or supplement the interpretation method such as quantitative survey. Additionally, the results may have been influenced by the cultural background, medical education curriculum, and environment of the medical college in which the study was conducted. There is a need to replicate this study on other cultures, curricula, or educational environments.

This study offers significant insights. First, it utilizes concept mapping analysis, allowing for a multidimensional understanding of happiness as medical students perceive it. This approach goes beyond traditional survey methods, capturing nuanced insights into students’ perceptions. Second, it categorizes happiness into distinct dimensions, such as personal development, social support, and professional fulfillment, providing actionable recommendations for improving students’ well-being.

### 4.2 Implications in medical education

Based on these research findings, we propose several interventions focusing on cultural and systemic factors that may influence the happiness of medical students. Medical schools should provide opportunities for mindfulness and emotional regulation training to enhance medical students’ positive psychological states. While physical activity and health showed similar correlations with happiness across different cultural contexts among medical students, it may be challenging to incorporate physical activity into the formal curriculum. However, it is necessary to create an educational environment and hidden curriculum that enables physical activity. Korean medical students appear to be characterized by collectivistic culture, Confucian values, and a social rather than individual self-concept. Therefore, medical schools should provide opportunities for students to develop a sense of belonging to their medical school and the community, while supporting the formation of social support networks. Although medical students face greater burden regarding examinations and academic workload compared with other majors, their future career security and stability influence how academic achievement affects their happiness. Notably, academic grades have a relatively minor impact on their experience of happiness, indicating a need for career guidance that can instill professional pride in their future careers, along with curriculum management that emphasizes the learning process rather than grades.

## 5 Conclusion

In this study, medical students recognized happiness based on two criteria: study and life, and self and relationships. The concept of happiness was categorized into “Personal development,” which was divided into “Self-management” and “Quality of life,” “Social support,” and “Professional fulfillment,” which comprised “Guaranteed future” and “Academic achievement.” Among these, “Social support,” “Self-management,” and “Quality of life” were considered to be the most important for medical students’ happiness.

Based on the results of this study, medical educational institutions and educators should support medical students’ happiness through emotional regulation and mindfulness programs, environments that support physical activities, programs that support positive social relationships, career and future coaching, and programs that support attitudes that emphasize the learning process over grades. Additionally, expanding on this research with a multicenter study with a larger sample size could enhance the results’ generalizability and applicability.

## Data Availability

The raw data supporting the conclusions of this article will be made available by the authors, without undue reservation.

## References

[1] Medvedev ON Landhuis CE. Exploring constructs of well-being, happiness and quality of life. Peer J. (2018) 6:e4903. doi: 10.7717/peerj.4903, PMID: 29876148 PMC5985772

[2] Shin DC Johnson DM. Avowed happiness as an overall assessment of the quality of life. Soc Indic Res. (1978) 5:475–92. doi: 10.1007/BF00352944

[3] Oishi S Diener E. Residents of poor nations have a greater sense of meaning in life than residents of wealthy nations. Psychol Sci. (2014) 25:422–30. doi: 10.1177/0956797613507286 24335603

[4] Yu NY Jeong YJ Kim BA Chong YS Shin HJ. An exploratory study on the concept of happiness in Korean undergraduate students. J Koreanol. (2015) 55:197–230. doi: 10.15299/jk.2015.5.55.197

[5] Layous K Lee H Choi I Lyubomirsky S. Culture matters when designing a successful happiness-increasing activity: a comparison of the United States and South Korea. J Cross-Cult Psychol. (2013) 44:1294–303. doi: 10.1177/0022022113487591

[6] Saleem S Saleem T. Role of religiosity in psychological well-being among medical and non-medical students. J Relig Health. (2017) 56:1180–90. doi: 10.1007/s10943-016-0341-5, PMID: 28028659

[7] Obregon M Luo J Shelton J Blevins T MacDowell M. Assessment of burnout in medical students using the Maslach burnout inventory-student survey: a cross-sectional data analysis. BMC Med Educ. (2020) 20:376. doi: 10.1186/s12909-020-02274-3, PMID: 33087080 PMC7579892

[8] Thun-Hohenstein L Hobinger-Ablasser C Geyerhofer S Lampert K Schreuer M Fritz C. Burnout in medical students. Neuropsychiatrie. (2021) 35:17–27. doi: 10.1007/s40211-020-00359-5, PMID: 32880881 PMC7954737

[9] Silva V Costa P Pereira I Faria R Salgueira AP Costa MJ. Depression in medical students: insights from a longitudinal study. BMC Med Educ. (2017) 17:184. doi: 10.1186/s12909-017-1006-0, PMID: 29017594 PMC5633876

[10] Rotenstein LS Ramos MA Torre M Segal JB Peluso MJ Guille C. Prevalence of depression, depressive symptoms, and suicidal ideation among medical students: a systematic review and meta-analysis. JAMA. (2016) 316:2214–36. doi: 10.1001/jama.2016.17324, PMID: 27923088 PMC5613659

[11] Hills P Argyle M. The Oxford happiness questionnaire: a compact scale for the measurement of psychological well-being. Pers Individ Dif. (2002) 33:1073–82. doi: 10.1016/S0191-8869(01)00213-6

[12] Rozman G. The East Asian region: Confucian heritage and its modern adaptation. Princeton, NJ: Princeton University Press (2014).

[13] Tan J Li H Zhang P Hirsh DA. Formed in context: a mixed-methods study of medical students' mindsets in an eastern culture. Med Educ. (2024) 59:210–25. doi: 10.1111/medu.15491 39166728

[14] Zhang XQ Zhang BS Wang MD. Application of a classroom-based positive psychology education course for Chinese medical students to increase their psychological well-being: a pilot study. BMC Med Educ. (2020) 20:323. doi: 10.1186/s12909-020-02232-z, PMID: 32962664 PMC7507630

[15] Yoo DM Kim DH. The relationship between students' perception of the educational environment and their subjective happiness. BMC Med Educ. (2019) 19:409. doi: 10.1186/s12909-019-1851-0, PMID: 31703671 PMC6839184

[16] Paulson BL Truscott K Stuart J. Client's perceptions of helpful experiences in counseling. J Couns Psychol. (1999) 46:317–24. doi: 10.1037/0022-0167.46.3.317

[17] Jeffrey A Johnsen JA Biegel DE Shafran R. Concept mapping in mental health: uses and adaptations. Eval Program Plann. (2000) 23:67–75. doi: 10.1016/S0149-7189(99)00038-5, PMID: 37783620

[18] Kane M Trochim WMK. Concept mapping for planning and evaluation. Thousand Oaks, CA: Sage Publications, Inc. (2007).

[19] Trochim WM McLinden D. Introduction to a special issue on concept mapping. Eval Program Plann. (2017) 60:166–75. doi: 10.1016/j.evalprogplan.2016.10.006 27780609

[20] Rosas SR Kane M. Quality and rigor of the concept mapping methodology: a pooled study analysis. Eval Program Plann. (2012) 35:236–45. doi: 10.1016/j.evalprogplan.2011.10.003, PMID: 22221889

[21] Jackson KM Trochim WMK. Concept mapping as an alternative approach for the analysis of open-ended survey responses. Organ Res Methods. (2016) 5:307–36. doi: 10.1177/109442802237114, PMID: 39917414

[22] Sturrock K Rocha J. A multidimensional scaling stress evaluation table. Field Methods. (2000) 12:49–60. doi: 10.1177/1525822X0001200104

[23] Shapiro SL Schwartz GE Bonner G. Effects of mindfulness-bas role of religiosity in psychological ed stress reduction on medical and premedical students. J Behav Med. (1998) 21:581–99. doi: 10.1023/A:1018700829825 9891256

[24] Zandi H Amirinejhad A Azizifar A Aibod S Veisani Y Mohamadian F. The effectiveness of mindfulness training on coping with stress, exam anxiety, and happiness to promote health. J Educ Health Promot. (2021) 10:177. doi: 10.4103/jehp.jehp_616_20, PMID: 34250111 PMC8249950

[25] Wu D Ye B Tang C Xue J Yang Q Xia F. Self-compassion and authentic-durable happiness during COVID-19 pandemic: the mediating role of meaning of life and the moderating role of COVID-19 burnout. Psychol Res Behav Manag. (2022) 15:3243–55. doi: 10.2147/PRBM.S380874, PMID: 36387037 PMC9642803

[26] Parkerson GR Jr Broadhead WE Tse CK. The health status and life satisfaction of first-year medical students. Acad Med. (1990) 65:586–8. doi: 10.1097/00001888-199009000-00009, PMID: 2400477

[27] Fisher JJ Kaitelidou D Samoutis G. Happiness and physical activity levels of first year medical students studying in Cyprus: a cross-sectional survey. BMC Med Educ. (2019) 19:475. doi: 10.1186/s12909-019-1790-9, PMID: 31888602 PMC6937866

[28] Rao R Naik BN Shekhar S Nirala SK Singh CM Verma M. Level of happiness among medical students in Bihar-an online survey. J Edu Health Promot. (2023) 12:305. doi: 10.4103/jehp.jehp_1806_22, PMID: 38023099 PMC10670860

[29] Yang X Wang M Wang J Zhang S Yang X Zhao L. Physical literacy and health of Chinese medical students: the chain mediating role of physical activity and subjective well-being. Front Public Health. (2024) 12:1348743. doi: 10.3389/fpubh.2024.1348743, PMID: 39056080 PMC11269216

[30] Doh YY Chung JB. What types of happiness do Korean adults pursue? Comparison of seven happiness types. Int J Environ Res Public Health. (2020) 17:1502. doi: 10.3390/ijerph17051502, PMID: 32110951 PMC7084433

[31] Monrouxe LV Chandratilake M Chen J Chhabra S Zheng L Costa PS. Medical students’ and trainees’ country-by-gender profiles: Hofstede’s cultural dimensions across sixteen diverse countries. Front Med. (2022) 8:746288. doi: 10.3389/fmed.2021.746288, PMID: 35211478 PMC8862177

[32] Kim EJ Lim JY Kim GM Kim SK. Nursing students' subjective happiness: a social network analysis. Int J Environ Res Public Health. (2021) 18:11612. doi: 10.3390/ijerph182111612, PMID: 34770124 PMC8583011

[33] Moghadam M Rezaei F Ghaderi E Rostamian N. Relationship between attachment styles and happiness in medical students. J Family Med Prim Care. (2016) 5:593–9. doi: 10.4103/2249-4863.197314, PMID: 28217589 PMC5290766

[34] Lin Y Kang YJ Lee HJ Kim DH. Pre-medical students’ perceptions of educational environment and their subjective happiness: a comparative study before and after the COVID-19 pandemic. BMC Med Educ. (2021) 21:619. doi: 10.1186/s12909-021-03065-0, PMID: 34911514 PMC8671600

[35] Kandi ZRK Ziapour A Pirouzeh R Faghihi M Jalilvand H Mansourian M. Survey of happiness in students of Iran University of Medical Sciences and its relationship with students' attitudes toward the field of education and the future of career. J Educ Health Promot. (2021) 10:439. doi: 10.4103/jehp.jehp_994_20, PMID: 35071645 PMC8719571

[36] Karavdic S Baumann M. Positive career attitudes effect on happiness and life satisfaction by master students and graduates. Open J Soc Sci. (2014) 2:15–23. doi: 10.4236/jss.2014.28003

[37] Tien Nam P Thanh Tung P Phuong Linh B Hanh Dung N Van Minh H. Happiness among university students and associated factors: a cross-sectional study in Vietnam. J Public Health Res. (2024) 13:22799036241272402. doi: 10.1177/22799036241272402, PMID: 39220811 PMC11365025

[38] Shin HI Jeon WT. "I'm not happy, but I don't care": help-seeking behavior, academic difficulties, and happiness. Korean J Med Educ. (2011) 23:7–14. doi: 10.3946/kjme.2011.23.1.7, PMID: 25814280 PMC8814484

[39] Park CH Kwon J Lee JT Ahn S. Impact of criterion versus norm-referenced assessment on the quality of life in Korean medical students. J Korean Med Sci. (2023) 38:e133. doi: 10.3346/jkms.2023.38.e133, PMID: 37128877 PMC10151616

